# Gadobutrol in India—A Comprehensive Review of Safety and Efficacy

**DOI:** 10.1177/1178623X17730048

**Published:** 2017-09-11

**Authors:** Jan Endrikat, Nicoletta Anzalone

**Affiliations:** 1 Radiology, Bayer AG, Berlin, Germany; 2 Department of Gynecology, Obstetrics and Reproductive Medicine, University Medical School of Saarland, Homburg, Germany; 3 Department of Neuroradiology, Scientific Institute HS Raffaele, Milan, Italy

**Keywords:** Magnetic resonance imaging, gadobutrol, safety, efficacy

## Abstract

Gadobutrol is a gadolinium (Gd)-based contrast agent for magnetic resonance imaging (MRI). In India, gadobutrol is approved for MRI of the central nervous system (CNS), liver, kidneys, breast and for MR angiography for patients 2 years and older. The standard dose for all age groups is 0.1 mmol/kg body weight. The safety profile has been demonstrated in 42 clinical phase 2 to 4 studies (>6800 patients), 7 observational studies, and by assessing pharmacovigilance data of 29 million applications. Furthermore, studies in children, adults, and elderly and in patients with impaired liver or kidney function did not show any increased adverse event rate. Diagnostic efficacy was demonstrated in numerous studies and various indications, such as diseases of the CNS, peripheral and supra-aortic vessels, kidneys, liver, and breast.

## Physicochemical Properties and Pharmacokinetics

Gadobutrol is a gadolinium (Gd)-based contrast agent (GBCA) for magnetic resonance imaging (MRI). In India, gadobutrol is approved in adults, adolescents, and children of 2 years and older, for MRI of the central nervous system (CNS), liver, kidneys, and breast and for MR angiography (MRA). The standard dose is 0.1 mL (=0.1 mmol) gadobutrol per kg body weight. For MRA, a fixed volume is recommended based on body weight and the number of fields of view (Table 1).^1^

**Table 1.** Gadobutrol—dosing.

Indications	Dosing	
	All weight classes	
CNS	0.1 mL/kg body weight (0.1 mmol/kg body weight)	
Liver		
Kidney		
Breast		
MRA	<75 kg body weight	≥75 kg body weight
Supra-aortal, aorta, abdominal, pelvic arteries (1 FOV)	7.5 mL	10 mL
	(0.1–0.15 mmol/kg body weight)	
Peripheral arteries (>2 FOVs)	15 mL	20 mL
	(0.2–0.3 mmol/kg body weight)	

Abbreviations: CNS, central nervous system; FOV, field of view; MRA, magnetic resonance angiography.

**Figure 1.**
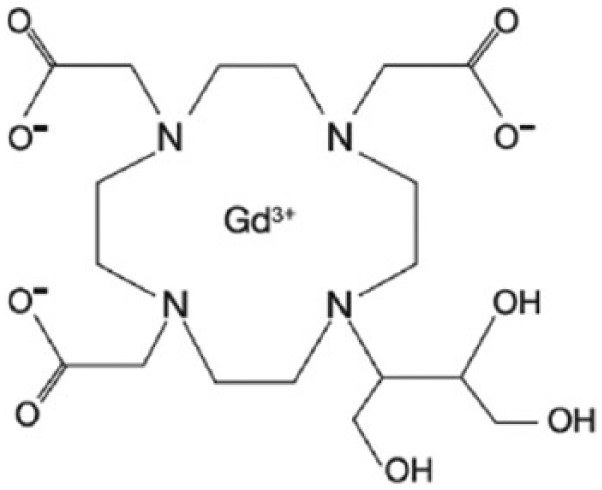
Molecular structure of gadobutrol.

Gadobutrol is a second-generation, multipurpose, nonionic extracellular, macrocyclic GBCA^2,3^ provided in a 1 M concentration (Figure 1). In addition to its unique 1 M concentration, gadobutrol features the highest relaxivity (the measure for the strength of a GBCA to shorten relaxation times) of all macrocyclic GBCAs^2,4,5^ (Table 2). The major determinant for signal and contrast enhancement in MRI is shortening of relaxation times of (water) protons. Due to gadobutrol’s high relaxivity and double concentration, it achieves the highest T1 shortening per mL of all GBCAs.^4^

**Table 2.** Gadobutrol—physicochemical data.

Parameter	Gadobutrol
Viscosity (at 37°C)	4.96 mPa s
Osmolality (at 37°C)	1603 mOsm/kg H_2_O
Partition coefficient (in *n*-octanol/buffer pH 7.6)	0.006
T1-relaxivity (r1) (37°C, 1.5 T) in plasma	5.2 (±0.3) L mmol^−1^ s^−1^
T2-relaxivity (r2) (37°C, 1.5 T) in plasma	6.1 (±0.3) L mmol^−1^ s^−1^
Thermodynamic complex stability	21.8 log Keq

Furthermore, as a macrocyclic contrast agent, gadobutrol provides high chelate stability with substantially less—if any—in vivo release of Gd ions as compared with linear GBCAs.^6^ The stability of Gd chelates has been linked to an increased risk of nephrogenic systemic fibrosis (NSF) in patients with severely impaired renal function.^7,8^ Because of these favorable characteristics, gadobutrol was categorized as a low-risk GBCA for development of NSF by several medical organizations^5,9^ and authorities.^10–12^

The clinical safety and efficacy of gadobutrol have been demonstrated in numerous clinical studies in children, adults, and elderly and will be reviewed here in detail.

## Safety

### Adverse events from clinical trials and postmarketing reports

The clinical trials program comprised 42 clinical phase 2 to 4 studies involving 6809 patients, including 184 children and adolescents aged <18 years. The incidence of drug-related adverse events (AEs) was 3.5% for gadobutrol and comparator GBCAs. All single drug–related AEs had an incidence of <0.5%, with the exception of nausea (0.7%). Hypersensitivity reactions were sporadic (<0.1%); however, patients with a history of allergies to contrast media experienced slightly more related AEs. The most frequent single drug–related AEs were headache, dysgeusia, and dizziness (Table 3).^13^ The postmarketing safety database comprises 29 million administrations as of December 2015 and confirms the safety profile shown in clinical studies (Table 4).^13^

**Table 3.** Related adverse events in clinical trials listed by MedDRA system organ classes.^13^

System organ class	Uncommon (≤0.7%)	Rare (≤0.1%)
Nervous system disorders	Headache Dysgeusia	Dizziness
Respiratory, thoracic, and mediastinal disorders		Dyspnea
Gastrointestinal disorders	Nausea	Vomiting
Skin and subcutaneous tissue disorders	Rash	Erythema Pruritus Paresthesia
General disorders and administration-site conditions	Feeling hot Injection-site reactions	Hypersensitivity

**Table 4.** Adverse drug reactions in postmarketing database.^13^

System organ class	Rare (<0.025%)
Immune system disorders	Anaphylactoid reactions^a^
Nervous system disorders	Dizziness, headache, tremor, loss of consciousness, convulsions, dysgeusia, hypoesthesia, sweating, vertigo
Gastrointestinal disorders	Nausea, vomiting, abdominal pain, difficulty swallowing, increased salivation
Eye disorders	Increased lacrimation
Cardiac disorders	Tachycardia, cardiac arrest
Vascular disorders	Hypertension, flushing, cyanosis, edema, syncope
Respiratory, thoracic, and mediastinal disorders	Dyspnea, throat/upper respiratory irritation, cough, chest pain, respiratory arrest, pulmonary edema, dysphonia
Skin and subcutaneous tissue disorders	Paresthesia, rash, dermatitis, pallor
General disorders and administration-site conditions	Feeling hot, malaise, injection-site reactions, feeling cold, burning sensation, skin reaction, pain/discomfort, asthenia

a Angioedema, anaphylactic/anaphylactoid reaction/shock, hypotension, bronchospasm, conjunctivitis, hypersensitivity reaction, erythema, rash, pruritus, laryngeal edema, sneezing, urticaria.

Forsting et al reported on 14 299 nonselected patients enrolled in 6 prospectively planned, observational surveillance studies in more than 300 institutions in Europe and Canada. In total, 78 of the 14 299 patients (0.55%) reported at least one adverse drug reaction (ADR). Two (0.01%) serious ADRs were recorded. Again, the most frequently reported ADR was nausea, which occurred in 36 patients (0.25%).^14^

In another most recent noninterventional prospective study by Prince et al, 23 708 unselected patients, including 1142 children, were enrolled to assess safety and tolerability of gadobutrol in routine practice. The overall rate of ADRs was 0.7%, those of serious AEs 0.02%. Most frequent ADRs were nausea (0.3%), vomiting, and dizziness (each 0.1%). The ADR rate was similar in patients with renal impairment or cardiac disease, from different geographic regions and in different gadobutrol dose groups. Patients at risk for contrast media reactions had an ADR incidence of 2.5%.^9^

All authors concluded that gadobutrol was well tolerated and has a favorable safety profile.

### Cardiovascular, hepatic, and renal tolerability

#### Heart

The cardiovascular tolerability of gadobutrol (0.1, 0.3, and 0.5 mmol/kg body weight) was evaluated in a randomized, double-blind, 5-times cross-over, placebo, and active (moxifloxacin) controlled study in 50 healthy volunteers. A positive effect on heart repolarization was demonstrated using 400 mg moxifloxacin. After gadobutrol administration, no participant experienced arrhythmias, palpitations, syncope, or seizures. In particular, patients with cardiovascular disease showed no greater risk of AEs. Overall, no relevant influence on heart rate, cardiac rhythm, pacing disturbances (extra-systoles), cardiac conduction or intervals (PQ, QRS, QT, including heart rate–corrected QT, ST, T wave) was recorded.^15^

#### Liver

Hepatic tolerability was evaluated by Voth et al.^16^ Patients with severe (more than 3 times upper limit of normal range [ULN]) and moderate (1.8 ≤3.0 times ULN) increase in liver enzymes (alanine aminotransferase and aspartate aminotransferase) showed an incidence of related AEs of 4.1% and 4.3%, respectively, compared with 5.1% in patients with normal liver function. These results suggest a similar safety profile in patients with hepatic impairment compared with the general population.^16^

#### Kidney

Initially, gadobutrol at doses from 0.1 up to 0.5 mmol/kg body weight was tested in 91 healthy volunteers in 2 phase 1 studies. The terminal half-life in plasma was approximately 1.5 hours. Total clearance approximated renal clearance, indicating glomerular filtration as the pathway of elimination. No metabolites were detected in plasma or urine up to 48 hours after injection. The renal excretion rate was linear over the large dose ranges tested, indicating dose-proportional, first-order kinetics. No change in urine chemistry, urinary enzymes, or creatinine clearance could be demonstrated.^17^

In 4 subsequent studies in patients with mild-to-severe renal impairment also, no trends for increased AEs were detected,^18–21^ although elimination half-life is prolonged in line with reduced estimated glomerular filtration rate (eGFR) in this population.^20^ Even in patients with marginal excretory function (creatinine clearance: <30 mL/min/1.73 m^2^), prehydration or treatment with diuretics or hemodialysis is not required after the administration of gadobutrol.^19,21^ In patients on hemodialysis, gadobutrol was dialyzable and could be removed from the blood almost completely (98%) within 3 dialysis sessions.^22^

### Tolerability in pediatrics and elderly

#### Children

Two studies specifically investigated the safety of gadobutrol in the age group of toddlers, children, and adolescents aged 2 to 18 years.^23,24^ Hahn et al recruited 138 patients undergoing routine MRI of the brain, spine, liver, kidneys, or MRA and assessed pharmacokinetics (PK) and safety of a single standard dose (0.1 mmol/kg body weight). They did not record any noteworthy changes in vital signs, cardiac rhythm, or oxygen saturation, neither any clinically significant change in renal laboratory parameters, serum creatinine, eGFR, total protein, albumin, blood urea nitrogen, or urine dipstick test. Within 6 hours after injection, 77% of administered dose was renally excreted. Eight patients (5.8%) experienced ADRs, including dysgeusia, feeling hot, crystallized urine (caused most probably by other medication), headache, nausea, rash, and pruritus.^23^ In a noninterventional, prospective, observational study, Glutig et al looked at safety of 1142 patients aged <18 years in the routine MRI setting. Rates of ADRs were low (0.5%), and no serious AEs were recorded. The ADRs did not show any correlation with pediatric age or gadobutrol weight-adjusted dose.^24^

Kuntze et al recruited 44 patients undergoing routine MRI of the brain, spine, liver, kidneys, or MRA and assessed PK and safety of a single standard dose (0.1 mmol/kg body weight). The PK profile was similar to that in older children and adults. In all, 1 of 44 patients (2.3%) experienced a drug-related AE (mild vomiting),^25^ whereas in a study of 57 patients <2 years, no AE was recorded.^26^

#### Elderly

Gadobutrol’s safety in the more fragile patient group of elderly, ie, patients aged >65 years, was assessed vs patients aged 18 to 64 years (“adults”) in 5608 patients from clinical studies, in 14 064 patients from postmarketing studies and ~12.7 million patients from pharmacovigilance reports.^27^ Overall, ADR rates were statistically significantly lower in elderly patients vs adults (in both clinical studies and in the pharmacovigilance population) due to a reduced incidence of nonserious ADRs. In the pharmacovigilance database, serious ADRs were reported in 334 (0.0038%) adults <65 years and in 87 (0.0022%) elderly patients. This comprehensive evaluation of data confirmed the favorable safety profile of gadobutrol, in general, and, in particular, in elderly patients.^27^

### Nephrogenic systemic fibrosis

As of December 31, 2016, a total of 13 reports of NSF or NSF-like symptoms in patients who reportedly were administered gadobutrol have been received. Five of these were “single-agent reports”; that is, in which patients reportedly received only gadobutrol.^13,28,29^ The other 8 reports were confounded by the administration of other GBCAs (“multiple-agent reports”). In assessing these reports, Bayer uses the criteria developed by Girardi et al^30^ and applies the criteria very conservatively. Not having direct access to the patient, the patient’s past contrast agent use, or even to the biopsy report in most cases, thus often having to rely on minimal information, Bayer gives the report the highest possible score based on the information available.^13^ Using this conservative “worst-case scenario” approach, 3 of the 5 single-agent reports meet the criteria for being diagnostic of or consistent with NSF^30^ and a possible association with gadobutrol cannot be excluded. The other single-agent reports contained information that was insufficient for evaluation. All 3 patients were multimorbid. The largest single dose administered to any patient with reported NSF was 0.49 mmol/kg body weight. Onset of NSF-like symptoms in these 3 reports occurred in 2006, 2008, and 2009. Onset latency ranged from 14 days to 18 months.^13^ A recent prospective multicenter study in 908 patients with moderate to severe renal impairment, ie, patients with increased risk for NSF, did not detect any case of NSF 2 years after gadobutrol-enhanced MRI.^31^

### Increased signal intensity and Gd presence in the brain

Since late 2013, reports were published on increased signal intensity (SI) and Gd presence in the brain (predominately globus pallidus and dentate nucleus) on unenhanced T1-weighted MRI scans after multiple administrations of mostly linear GBCAs. Twelve clinical studies investigated gadobutrol, a macrocyclic GBCA. Of these, 9 studies^32–40^ did not show increased SI, 3 presented mixed results.^41–43^ One paper by Stojanov et al^41^ reported to have seen increased SI after gadobutrol administration. However, the study design and evaluation was criticized by Agris et al.^44^ One preclinical study by Jost et al^45^ did not find increase in SI after gadobutrol administration. Apart from increased SI, Murata et al^46^ reported on Gd presence in the brain of linear and macrocyclic GBCAs, including gadobutrol.

## Efficacy

### Central nervous system

There are a number of clinical head-to-head studies comparing gadobutrol with other GBCAs.

The initial studies were conducted vs gadopentetate by Anzalone et al^47^ and Kim et al.^48^ Anzalone et al^47^ reported improved lesion conspicuity for gadobutrol in 10/27 (37%) patients with brain metastases in an intraindividual comparison. Although in the remaining 17 patients conspicuity was equivalent, in 2 patients, a lesion was only seen with gadobutrol. Similarly, Kim et al compared double doses of both GBCAs in 27 patients with brain metastases and detected 25/155 lesions only with gadobutrol. In addition, the mean contrast-to-noise ratio (CNR) was higher on equivalent gadobutrol images than on gadopentetate images (2.17 ± 0.19 vs 1.90 ± 0.26; *P* = .00011).^48^

A multicenter, randomized study with 3 blinded readers compared gadobutrol with gadoterate intraindividually in 136 patients with brain tumors. Superiority of gadobutrol over gadoterate for overall preference was demonstrated in 131/199 (65.8%) patients. Furthermore, significantly better lesion contrast and relative lesion enhancement were recorded.^49^

Furthermore, 2 large phase 3 studies evaluated gadobutrol’s efficacy in brain imaging vs gadoteridol. Katakami et al enrolled 175 patients with brain metastases to assess efficacy and safety of 2 doses of gadobutrol (0.1 and 0.2 mmol/kg body weight) in comparison with double dose of gadoteridol (0.2 mmol/kg body weight). They showed single dose of gadobutrol to be noninferior to a double dose of gadoteridol at detecting brain metastases.^50^ Gutierrez et al^51^ performed a phase 3 study with 390 patients concluding that gadobutrol demonstrates greater contrast enhancement and improved sensitivity and accuracy for detection of malignant disease than gadoteridol, likely because of its higher relaxivity.

Finally, gadobutrol is also recommended for CNS perfusion imaging, as it displays a sharper bolus peak and increased first-pass concentration than 0.5 M agents.^52^

**Figure 2.**
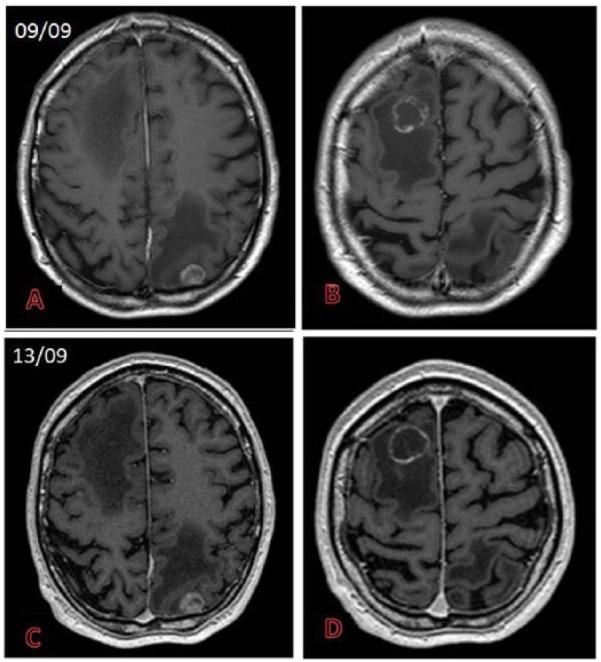
Multiple brain metastasis from lung cancer with ring-like enhancement and surrounding edema (A-D: postcontrast T1-weighted sequences) in the right frontal and left occipital lobes studied within an interval of 4 days (September 9 and September 13) with different contrast agents ( 13 mL of gadoterate meglumine in A and B and 6.5 mL of gadobutrol in C and D), with the same postcontrast delay. Better and more consistent enhancement seen in C and D due to the higher relaxivity of the contrast agent.

**Figure 3.**
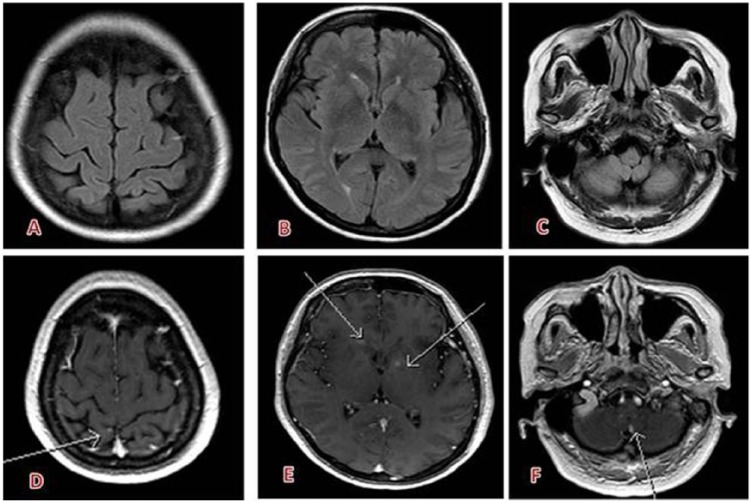
Multiple small brain metastases (breast cancer) visible only on T1-weighted sequences after intravenous contrast injection (gadobutrol, 7.5 mL) (D) right parietal lesion, (E) left lenticular and right head of caudate lesions, (F) left cerebellar lesion and not on (A-C) fluid-attenuated inversion recovery sequence.

**Figure 4.**
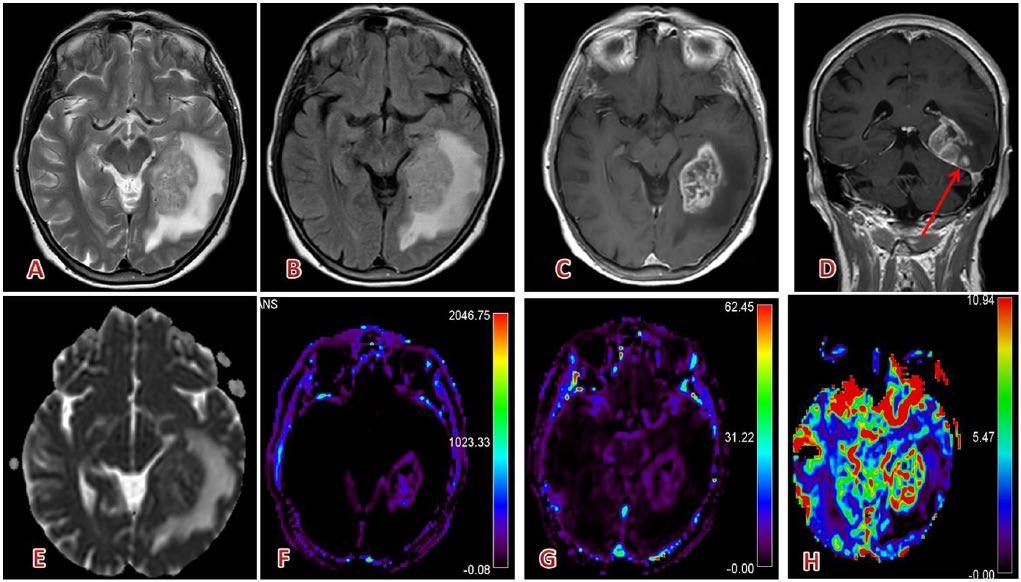
Typical glioblastoma (WHO grade IV): (A) left temporal big lesion, inhomogeneous and slightly hyperintense on T2 and (B) fluid-attenuated inversion recovery, with high, irregular enhancement after gadolinium-based contrast agent injection (gadobutrol: 5 + 5mL according to perfusion protocol in (C) and (D)), necrotic components, and restricted diffusion (apparent diffusion coefficient map, E); surrounding edema (A, B, E), small satellite lesion (arrow in D); high DSC and DCE perfusion parameters (Ktrans: F, Vp: G, rCBV: H).

Examples for CNS imaging with gadobutrol are given in Figures 2 to 4.

### Angiography

#### Peripheral MRA, peripheral arterial occlusive disease

Three publications focused on the assessment of pelvic and peripheral arteries comparing gadobutrol MRA vs intra-arterial digital subtraction angiography (DSA). Hentsch et al^53^ prospectively investigated 203 patients with peripheral arterial occlusive disease (PAOD) and a sensitivity of 93% and a specificity of 90% for detection of clinically significant stenosis in the on-site evaluation. Similar results for whole-body MRA were found by Herborn et al with overall sensitivities of 92% to 93% and specificities of 87% to 89% (2 readers). They summarize that gadobutrol-enhanced MRA permits a rapid, noninvasive, and accurate evaluation of the lower peripheral arterial system in patients with PAOD.^54^ These results were recently confirmed by Loewe et al^55^ in 156 patients with PAOD in comparison with gadoterate and DSA as standard of reference.

#### Cerebral vessels

**Figure 5.**
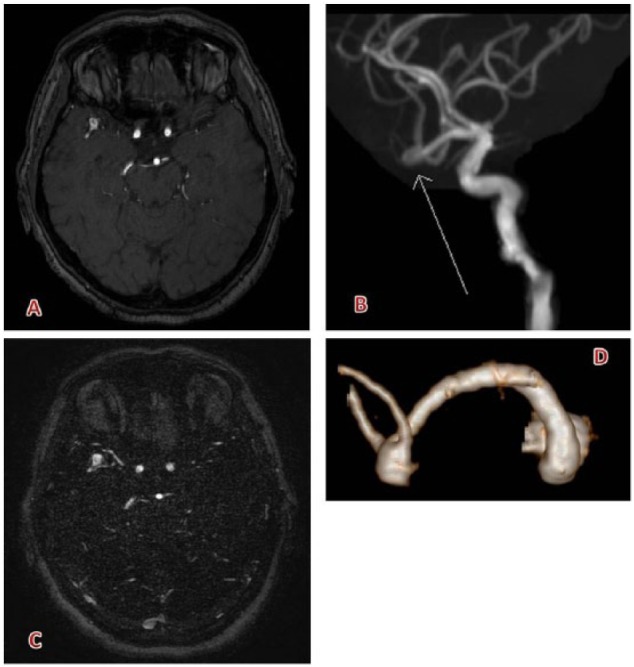
Small aneurysm (6 mm) of the right middle cerebral artery bifurcation: (A) 3D-time-of-flight sequence and (B) relative maximum intensity projection reconstruction, (C) 3D-contrast-enhanced magnetic resonance angiography, and (D) relative 3D surface rendering which allows a better delineation of aneurysm’s morphology. Contrast agent: gadobutrol (7 mL). 3D indicates 3-dimensional.

Visualization of supra-aortic vessels, ie, proximal and distal internal carotid arteries, was investigated by Kramer et al in 22 healthy volunteers in a blinded, prospective, randomized, intraindividual comparison of gadobutrol, gadobenate, and gadoterate. Signal-to-noise ratio (SNR) in static MRA was significantly higher for gadobutrol vs both other GBCAs (*P* < .05). Pairwise qualitative overall preference analysis showed gadobutrol superior to gadobenate in 10 (50%) and to gadoterate in 17 (85%) of volunteers. The authors conclude that for MRA of the carotid arteries, 1.0 M gadobutrol shows higher image quality and higher SNR and CNR as compared with 0.5 M GBCAs.^56^ An example of cranial vessel angiography is shown in Figure 5.

#### Whole-body MRA

Magnetic resonance angiography with gadobutrol from head to toe was performed by Schaefer et al in 179 patients with a broad range of vascular diseases and indications of vessel assessment. The agreement between MRA and DSA diagnosis was statistically significant in the on-site (96.6%) and blinded reader (86.6%-90.2%) evaluation. Sensitivity, specificity, accuracy, positive predictive value (PPV), and negative predictive value (NPV) for detection of relevant stenosis (>50%) were calculated for the right and left internal carotid arteries and common and external iliac arteries: on-site reading sensitivity was 95% to 98%, specificity 94% to 96%, accuracy 96%, NPV 98% to 99%, and PPV 79% to 93%. Gadobutrol-enhanced MRA of body arteries provides diagnostic information comparable with intra-arterial DSA.^70^ Hadizadeh et al provided evidence that the visualization of individual vessel segments is significantly better after administration gadobutrol compared with gadopentetate (*P* < .001).^57^

### Kidney and liver

#### Kidney

**Figure 6.**
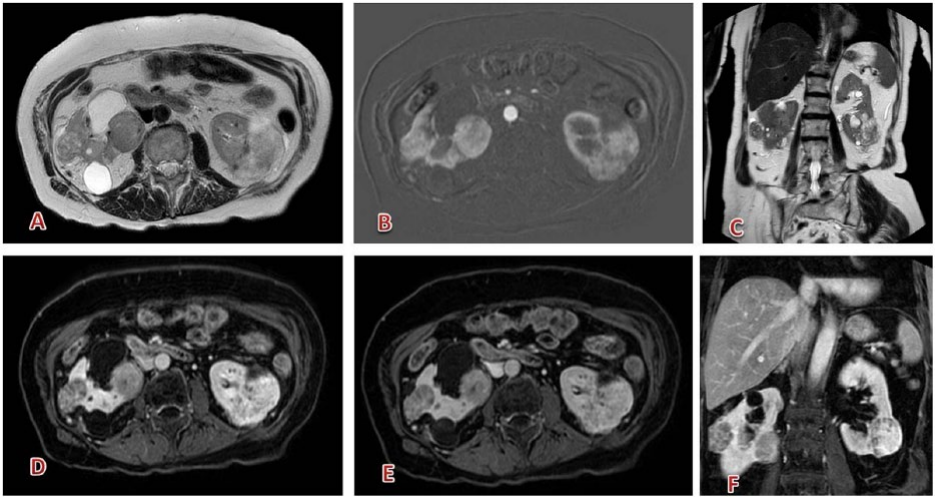
Multiple renal oncocytomas: (A, C) slightly hyperintense on T2, with hypointensity spots inside (central scar); (D, F, and B) moderate enhancement in arterial phase (subtraction); (E) progressive late washout (portal phase). Contrast agent: gadobutrol, 8 mL. Courtesy of Professor De Cobelli, Radiology Department, San Raffaele Scientific Institute, Milan.

**Figure 7.**
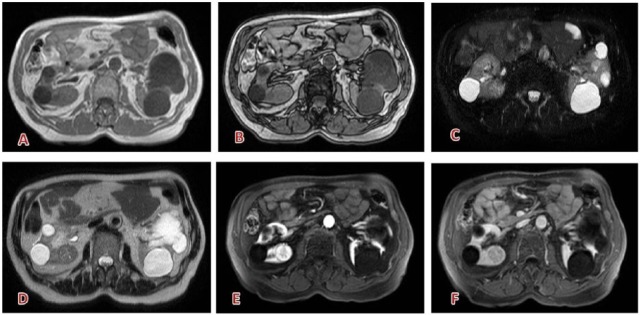
Typical clear-cell carcinoma of the right kidney: well-defined capsulated solid lesion, (A) hypointense on T1-weighted, with subtle signal loss in the out-of-phase sequence (intracellular fat, B); (C) partially restricted diffusion, inhomogeneous; (D) slightly hyperintense on T2; (E) strong and rapid enhancement in corticomedullary phase; (F) best seen on nephrogenic phase. Contrast agent: gadobutrol, 6.5 mL. Courtesy of Professor De Cobelli, Radiology Department, San Raffaele Scientific Institute, Milan.

A large multicenter, randomized study assessed the efficacy of gadobutrol vs gadopentetate in 471 patients with known or suspected focal renal lesions in an interindividual design. Standard of reference was contrast-enhanced computed tomography and 3 independent blinded readers—summarized as the “average reader”—interpreted the images. The diagnostic accuracy of the average reader was 83.7% for gadobutrol and 87.3% for gadopentetate. The increase in accuracy from precontrast to combined pre- and postcontrast MRI was 8.0% for gadobutrol and 6.9% for gadopentetate. Sensitivity for gadobutrol and gadopentetate was 85.2% and 88.7%, respectively, and specificity was 82.1% and 86.1%, respectively.^58^ Artunc et al introduced a new aspect of kidney MR with gadobutrol. They described an MR-based approach to comprehensively evaluate both kidney anatomy and function, MR-glomerular filtration rate, in a single investigation.^59^ Examples for contrast-enhanced kidney imaging are shown in Figures 6 and 7.

#### Liver

**Figure 8.**
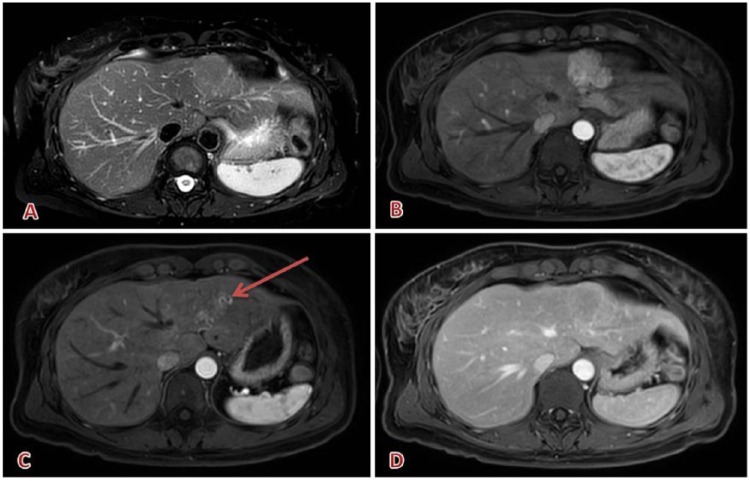
Typical hepatic focal nodular hyperplasia: (A) iso-hyperintense on T2, (B) early and strong washin in arterial phase; (D) rapid washout (portal phase); (C) serpiginous arterial vessels inside the lesion (arrow). Contrast agent: gadobutrol, 7 mL. Courtesy of Professor De Cobelli, Radiology Department, San Raffaele Scientific Institute, Milan.

**Figure 9.**
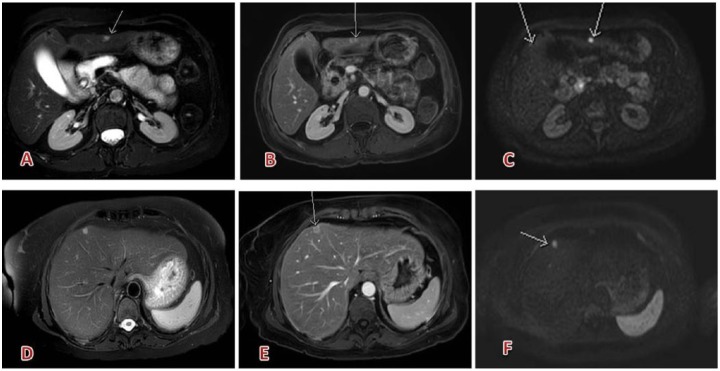
Presence of 2 hepatic metastasis from colon carcinoma: one in the left lobe and the other in the IV segment. These lesions are hyperintense on (A, D) T2; (B, E) early arterial enhancement; (C, F) restricted diffusion. Contrast agent: gadobutrol, 7 mL. Courtesy of Professor De Cobelli, Radiology Department, San Raffaele Scientific Institute, Milan.

Also liver imaging with gadobutrol was assessed in a large, randomized interindividual phase 3 study in 572 patients with liver lesions vs gadopentetate. After administration of gadobutrol, combined pre- and postcontrast MRI increased accuracy by 19.9%, sensitivity by 33.0%, and specificity by 8.5%. The authors finally claim noninferiority of gadobutrol to gadopentetate in the diagnostic assessment of liver lesions.^60^ Examples of liver imaging are shown in Figures 8 and 9.

### Breast

There are 3 seminal studies investigating gadobutrol in breast MRI. Pediconi et al compared gadobutrol vs gadobenate in a multicenter, prospective, intraindividual study in 72 patients. They found sensitivities of gadobutrol for lesion detection of 82.3% and for lesion characterization of 92.6%. The figures for gadobenate were very similar. Almost two-thirds of the readers were very confident and one-third confident with the images.^61^ Another study by Fallenberg et al assessed gadobutrol vs gadoterate in an intraindividual, randomized comparison in 52 women with benign or malignant breast lesions. Primary end point was the relative enhancement of the dynamic imaging. Mean relative enhancement was significantly higher for gadobutrol than for gadoterate (*P* < .0001) and also peak enhancement was higher, likely due to gadobutrol’s higher relaxivity.^62^ The largest body of data on gadobutrol in breast MRI was reported by Sardanelli et al. They provide data on 2 large phase 3 studies including 787 women with proven breast cancer. Sensitivity ranged from 80% to 89% for gadobutrol MRI and was significantly superior to mammography (68%-73%). Specificity for MRI ranged from 83% to 95%.^63^ Table 5 shows data on index cancer detection and detection of additional lesions specifically from India and from all over the world. Although taken the small sample size into account, the subevaluation of Indian patients was in line with the global results showing higher detection rates for breast MRI compared with mammography.

**Table 5.** Detection of index cancer or additional cancer with gadobutrol-enhanced breast MRI or mammography in the GEMMA 2 study: Indian vs global results.^63^

	Reader	India		Global	
		Patients	%	Patients	%
Imaging method					
A. Proportions of patients whose index cancer was detected^a^					
Breast MRI	1	58	96.6	388	89.2
	2	58	96.6	388	88.9
	3	58	96.6	388	85.6
	Investigator	58		388	99.0
Mammography	1	58	77.6	388	69.3
	2	58	84.5	388	75.0
	3	58	87.5	388	72.7
	Investigator	58		388	96.6
B. Proportion of patients where at least 1 additional cancer was detected					
Breast MRI	1	12	80.0	58	69.0
	2	13	86.7	66	78.6
	3	12	80.0	57	67.9
	Investigator	14	93.3	61	72.6
Mammography	1	6	40.0	22	26.2
	2	5	33.3	25	29.8
	3	7	46.7	35	41.7
	Investigator	6	40.0	27	32.1

Abbreviation: MRI, magnetic resonance imaging.

a Index cancer is defined as a malignancy which made the patient eligible for the trial and which was confirmed by the reference standard. Per-patient detection of index cancer shows the proportion of patients where all index cancers were detected.

**Figure 10.**
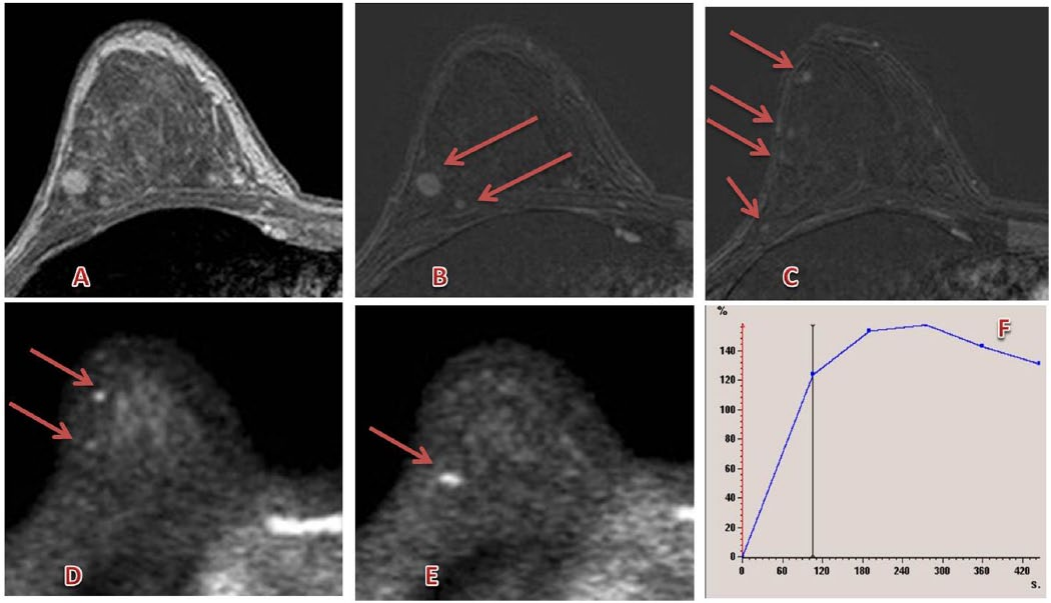
Multicentric left breast lesion (the greater of about 3.5 cm) between upper quadrants with irregular lobulated margins, restricted diffusion, and early and strong enhancement (lobular carcinoma). (A, B) T2 images; (C, D) diffusion-weighted images; (E-G) postcontrast subtracted images; and (H) enhancement curve. Contrast agent: gadobutrol, 9 mL. Courtesy of Professor De Cobelli, Radiology Department, San Raffaele Scientific Institute, Milan.

**Figure 11.**
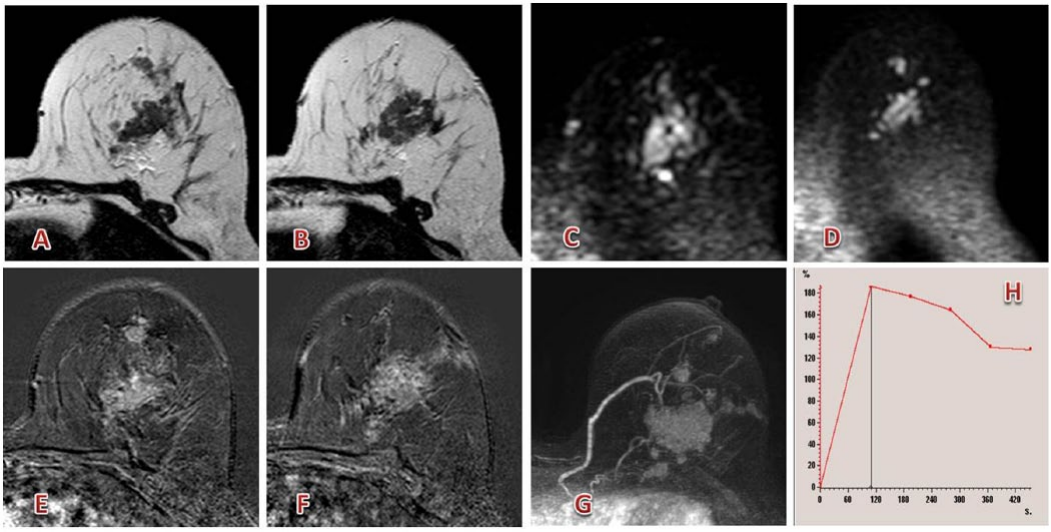
Multicentric right breast lesion (greater than ~1 cm) in the upper-outer quadrant with irregular margins, restricted diffusion, and strong enhancement (infiltrative ductal carcinoma). (A-C) Postcontrast and subtracted images; (D, E, F, G) diffusion-weighted images; (H) enhancement curve. Contrast agent: gadobutrol, 8 mL. Courtesy of Professor De Cobelli, Radiology Department, San Raffaele Scientific Institute, Milan.

Sardanelli et al^63^ concluded their very large multicenter preoperative setting, gadobutrol-enhanced breast MRI demonstrated high levels of sensitivity and specificity, consistent with published data on breast MRI. Examples for breast MRI are shown in Figures 10 and 11.

## Discussion

### Physicochemical properties and PK

As of today, gadobutrol is approved in India for MRI of the CNS, liver, kidney, breast and for MRA in adults and children (2 years of age and older). This is different in other regions, eg, in Europe, most of the countries in South America, the Middle East, Asia, and Australia gadobutrol is approved for all body regions and all age groups (including neonates). Gadobutrol features the combination of high relaxivity and 1 M concentration.

### Safety

#### General safety

General safety of gadobutrol has been assessed in 6809 patients from 42 clinical phase 2 to 4 studies,^13^ in 7 prospective observational studies,^9,14^ and by analyzing pharmacovigilance data comprising 29 million applications.^13^ However, a direct safety comparison of different GBCAs is challenging, as no head-to-head prospective studies are available. Instead, putting phase 4 studies side by side might be an option. So, overall ADR rates were reported for gadobutrol,^14^ gadopentetate,^64^ gadoterate,^65^ and gadobenate^66^ of 0.55%, 2.4%, 0.4%, and 0.76%, respectively. Nausea and vomiting were always the most frequently reported ADRs. Overall, the safety profile and tolerability of the investigated GBCAs were similar.

#### Cardiovascular, liver, and renal safety

Specific studies were conducted to investigate gadobutrol’s potential impact on the heart and its tolerability in patients with liver or kidney diseases. Even up to 0.5 mmol/kg body weight, ie, 5-times standard dose, no effect on heart parameters was detected.^15^ Also, in patients with elevated liver enzymes, the incidence of AEs was similar to patients with normal liver function.^16^ Although gadobutrol is excreted exclusively via the kidneys, no increase in AE incidence was observed in patients with mild-to-severe renal impairment.^18–21^

#### Safety in children and elderly

The safety of gadobutrol was investigated intensively in the “most vulnerable age groups,” ie, children (including neonates) and patients ≥65 years of age. While Hahn et al^23^ looked at children aged 2 to 17 years in a clinical phase 2 setting, Glutig et al^24^ took the more real-life perspective of an observational trial, including children aged younger than 2 years as well. A specific study on PK and safety of gadobutrol in children aged younger than 2 years including term newborns was performed by Kunze et al.^25^ They investigated 44 children; of them 9 term newborns and infants aged <2 months. They confirmed the favorable safety profile seen in children <2 years and concluded that the safety in this very young age group was similar to older children and adults. They summarized that the recommended standard dose is also appropriate in children aged younger than 2 years.^25^ Finally, Bhargava et al^26^ also focused on patients <2 years in a single-center observational study. They did not detect any AE related to gadobutrol in 57 patients. Similar low AE rates were also found for gadobutrol, gadopentetate, and gadobenate in a recent retrospective review of 2393 children by Neeley et al.^67^

Unique data were collected for elderly patients, ie, patients aged >65 years.^27^ This group is of particular clinical importance as in many countries the population is rapidly aging and this group will constitute an increasing share of the patients in the MR suite. To the best of our knowledge, no such data had been published for other GBCAs, although a similar study is running on gadoxetic acid. Here, a comparable favorable AE profile is seen in elderly compared with adults.^68^

#### Nephrogenic systemic fibrosis

As of December 31, 2016, 3 so-called “unconfounded” or single-agent reports of patients with NSF-like symptoms were received for which a possible association with gadobutrol cannot be excluded.^13^ As noted previously, Bayer always applies the most conservative approach when assessing these reports and this conservative assessment combined with other factors, including market share, date of market entry, and variability in interpretation of the data (and even in interpretations of terms such as “unconfounded”), may influence the number of reports. Bayer considers another GBCA as a plausible confounding factor if it was administered within 18 months of the Bayer product and before NSF onset. Products administered 10 years earlier, therefore, would not plausibly be considered confounders. No new reports on NSF with onset after 2009 have been received concerning gadobutrol.

#### Increased SI and Gd presence in the brain

With respect to increased SI and Gd presence in the brain, scientific knowledge is still evolving. It seems that primarily linear and not macrocyclic GBCAs are associated with SI increase in the brain.^32,35–37^ So far, no clinical symptoms or adverse health effects associated with this increased SI have been confirmed in the literature or in pharmacovigilance databases. On July 21, 2017, the European Medicines Agency’s Committee for Medicinal Products for Human Use published its opinion on Gd presence in brain, confirming the macrocyclics’ higher stability and lower propensity to release Gd compared with linear agents.^69^

### Efficacy

#### Central nervous system

A plethora of studies, including prospective head-to-head studies, have been performed on efficacy. For contrast-enhanced CNS, MRI publications of gadobutrol vs gadopentetate,^47,48^ gadoterate,^49^ and gadoteridol^50,51^ are available. In all studies, certain efficacy parameters were superior to the comparators, be it conspicuity,^39^ CNR,^40^ overall preference and lesion contrast/enhancement,^49^ or improved sensitivity and accuracy for detection of malignant lesions likely due to the high relaxivity.^51^

#### Magnetic resonance angiography

Magnetic resonance angiography studies were run in patients with PAOD, stenotic cerebral vessels, or in need for a whole-body angiography. For PAOD, a high sensitivity and specificity comparable with DSA was shown.^53,55,65^ As MRA is noninvasive and does not apply ionizing radiation, MRA could be seen as a clinical alternative procedure to invasive intra-arterial DSA. Kramer et al^56^ showed higher image quality and higher SNR and CNR for gadobutrol vs 2 other GBCAs in visualization of supra-aortic vessels. However, this study was in 22 healthy volunteers and it is not clear whether these results also would apply for patients with severe atherosclerotic carotids. Also, for whole-body MRA, gadobutrol-enhanced imaging showed high sensitivity, specificity, accuracy, NPV, and PPV compared with DSA.^70^

#### Kidney, liver, and breast

In one clinical phase 3 study, the clinical utility for kidney imaging with gadobutrol has been shown. There is a clear benefit of combining pre- and postcontrast MRI vs precontrast MRI only. However, a significant advantage of gadobutrol vs gadopentetate could not be shown.

Gadobutrol can also be used for liver imaging. However, as a pure extracellular GBCA, no information on liver cell function can be gathered. Gadoxetic acid, a liver-specific GBCA, might be a better option.^60^

Three publications reported on the usage of gadobutrol in breast MRI,^61–63^ all showing promising data on detecting and characterizing breast lesions. Interestingly, Sardanelli et al^63^ showed a higher sensitivity of breast MR vs x-ray mammography, whereas specificity was in a comparable range. Their findings confirm previous results by Kuhl et al and Leach et al who reported sensitivities of breast MR vs x-ray mammography of 90.7% and 77% vs 32.6% and 40%, respectively. Specificities for both modalities were above 90%.^71,72^ American and European guidelines, however, see x-ray mammography as first-line imaging modality and recommend breast MRI for certain clinical situations in screening (high-risk patients, contralateral breast) and preoperative staging (multifocality, multicentricity, invasion in fascia, lobular cancer, and discrepancy between x-ray and ultrasound).^73,74^

## Conclusions

Gadobutrol, provided at unique 1 M concentration, is a safe and effective macrocyclic GBCA for MRI recommended for a broad range of clinical indications and age groups.
